# Transcriptome Analysis to Identify Cold-Responsive Genes in Amur Carp (*Cyprinus carpio haematopterus*)

**DOI:** 10.1371/journal.pone.0130526

**Published:** 2015-06-22

**Authors:** LiQun Liang, YuMei Chang, XuLing He, Ran Tang

**Affiliations:** 1 National & Local United Engineering Laboratory of Freshwater Fish Breeding, Key Laboratory of Freshwater Aquatic Biotechnology and Genetic Breeding, Ministry of Agriculture, Key Laboratory of Fish Stress Resistance Breeding and Germplasm Characteristics on Special Habitats, Heilongjiang River Fisheries Research Institute, Chinese Academy of Fishery Sciences, Harbin, Heilongjiang, P. R. China; 2 Chinese National Human Genome Center at Shanghai, Shanghai, P. R. China; Institute of Hydrobiology, Chinese Academy of Sciences, CHINA

## Abstract

The adaptation of fish to low temperatures is the result of long-term evolution. Amur carp (*Cyprinus carpio haematopterus*) survives low temperatures (0-4°C) for six months per year. Therefore, we chose this fish as a model organism to study the mechanisms of cold-adaptive responses using high-throughput sequencing technology. This system provided an excellent model for exploring the relationship between evolutionary genomic changes and environmental adaptations. The Amur carp transcriptome was sequenced using the Illumina platform and was assembled into 163,121 cDNA contigs, with an average read length of 594 bp and an N50 length of 913 bp. A total of 162,339 coding sequences (CDSs) were identified and of 32,730 unique CDSs were annotated. Gene Ontology (GO), EuKaryotic Orthologous Groups (KOG) and Kyoto Encyclopedia of Genes and Genomes (KEGG) analyses were performed to classify all CDSs into different functional categories. A large number of cold-responsive genes were detected in different tissues at different temperatures. A total of 9,427 microsatellites were identified and classified, with 1952 identifying in cold-responsive genes. Based on GO enrichment analysis of the cold-induced genes, “protein localization” and “protein transport” were the most highly represented biological processes. “Circadian rhythm,” “protein processing in endoplasmic reticulum,” “endocytosis,” “insulin signaling pathway,” and “lysosome” were the most highly enriched pathways for the genes induced by cold stress. Our data greatly contribute to the common carp (*C*. *carpio*) transcriptome resource, and the identification of cold-responsive genes in different tissues at different temperatures will aid in deciphering the genetic basis of ecological and environmental adaptations in this species. Based on our results, the Amur carp has evolved special strategies to survive low temperatures, and these strategies include the system-wide or tissue-specific induction of gene expression during their six-month overwintering period.

## Introduction

Thermal tolerance determines the habitat selection, spatial distribution and migration of wild fish and affects the survival, growth, reproduction, and productivity of farmed fish [1–3]. Thus, water temperature is proposed to be the master abiotic factor that affects all life activities of fish. Faced with daily and seasonal temperature fluctuations, fish have evolved adaptive responses to the stress of temperature fluctuation [4, 5]. Of the approximately 28,000 species of fish, some occupy well-defined temperature niches, which rang from −1.5°C at the poles to 45°C in hot springs [5]. Despite this broad temperature range, many fish are killed in the wild and on farms by winter cold fronts, which are particularly problematic for commercially important aquaculture species that are sensitive to cold stress, such as tilapia (*Oreochromis niloticus*), milkfish (*Chanos chanos*), cobia (*Rachycentron canadum*) and red sea bream (*Pagrus major*) [6–8]. The annual economic losses from the mass mortality caused by exposure to low temperatures are devastating to regions and countries that depend on aquaculture, including Taiwan, China, Israel and South America [7]. Therefore, for the purpose of advancing scientific research and increasing the sustainability of fisheries, it is essential to investigate the mechanisms of cold tolerance in fish.

Poikilothermic (cold-blooded) animals have developed several biochemical and physiological adaptations to survive exposure to diurnal or seasonal low temperatures and chronic cold. In Antarctic notothenioids, adaptive changes for cold survival have been reported in various studies. One striking discovery was the identification of antifreeze glycoprotein (AFGP), which is considered to be one of the major factors involved in the expansion of populations of polar fish into harsh environments [9]. Another achievement was the discovery that many genes have been lost, including hemoproteins in the icefish family Channichthyidae, likely as the result of an evolutionary response to chronic cold [10]. Unlike polar fish species, eurythermal fish such as the common carp (*Cyprinus carpio*) use different strategies to survive low temperatures. One famous hypothesis regarding the mechanism of survival at cold temperature is “homeoviscous adaptation,” which suggests that fish can avoid freezing by decreasing the saturation of membrane phospholipids to offset the cold-induced rigidification of lipid bilayers during cold periods [11].

With the rapid development of high-throughput sequencing technology, a large number of genes associated with cold tolerance have been identified using microarray or RNA sequencing techniques in different species of fish, including channel catfish (*Ictalurus punctatus*) [12], common carp [13], Antarctic notothenioid fish [14, 15], zebrafish (*Danio rerio*) [16–19] and tilapia [6, 20]. These genes are involved in a wide range of biological processes, including transcriptional regulation, genomic expansion, protein homeostasis, cell cycle control, cytoskeletal reorganization, signal transduction, cell growth and differentiation, stress responses and metabolic regulation. Previous studies have demonstrated that cold tolerance is a complex trait under the control of multiple genes that trigger a complex gene expression pattern during cold stress.

Common carp has a wide thermal range, it tolerates winter temperatures close to 0°C and survives at 30°C in tropical ponds. Additionally, rapid cold challenge tests have indicated that common carp can respond to much larger thermal changes and can become habituated to rapid temperature shocks [21]. Thus, many studies have used common carp as an experimental model to investigate fish cold tolerance-related issues [11, 13, 22, 23]. However, clear differences in cold tolerance have been found among different populations or subspecies of overwintering common carp [24]. Moreover, fish adaptation to low temperatures is the result of long-term evolution. Amur carp (*Cyprinus carpio haematopterus*) inhabits the Amur River, which is located in the northern region between Russia and China. The cold air from Siberia affects this region year-round, and Amur carp must survive low temperatures (0–4°C) for six months. Therefore, the Amur carp system is very useful for studying the mechanisms of adaptation to cold and for exploring the relationship between evolutionary genomic change and environmental adaptation.

The physiological functioning of fish is directly and critically affected by ambient temperatures [25], and certain physiological properties must be activated for acclimatization to the cold [7]. Previous studies of cold stress in fish have focused primarily on molecular responses to hours or up to a few days of acclimation and made comparisons of low and high temperature treatments. In order to fully understand the evolution mechanisms of Amur carp that is how to adapt to the long-term low temperature stress, we investigated temperature compensation mechanisms following long-term cold acclimation with graded cooling temperatures in Amur carp based on its own biological characteristics and life history. Therefore, we compared the transcriptome profiles in different tissues at different temperatures (20°C, 13°C and 5°C) with the same period of acclimation (15 days) to determine the following: (1) genes that are most affected during extended exposure to lower temperatures and (2) genes that show a marked increase in expression as the temperature decreased (i.e., cold-responsive genes). Notably, we found that Amur carp evolved special long-term strategies to survive low temperatures and that these strategies included the system-wide and tissue-specific induction of gene expression (particularly of immune response-related genes), and specific induction of cold-responsive genes during an overwintering period of six months.

## Results and Discussion

### Generation and *De novo* Assembly of Amur Carp Transcriptome Data

**Fig 1:**
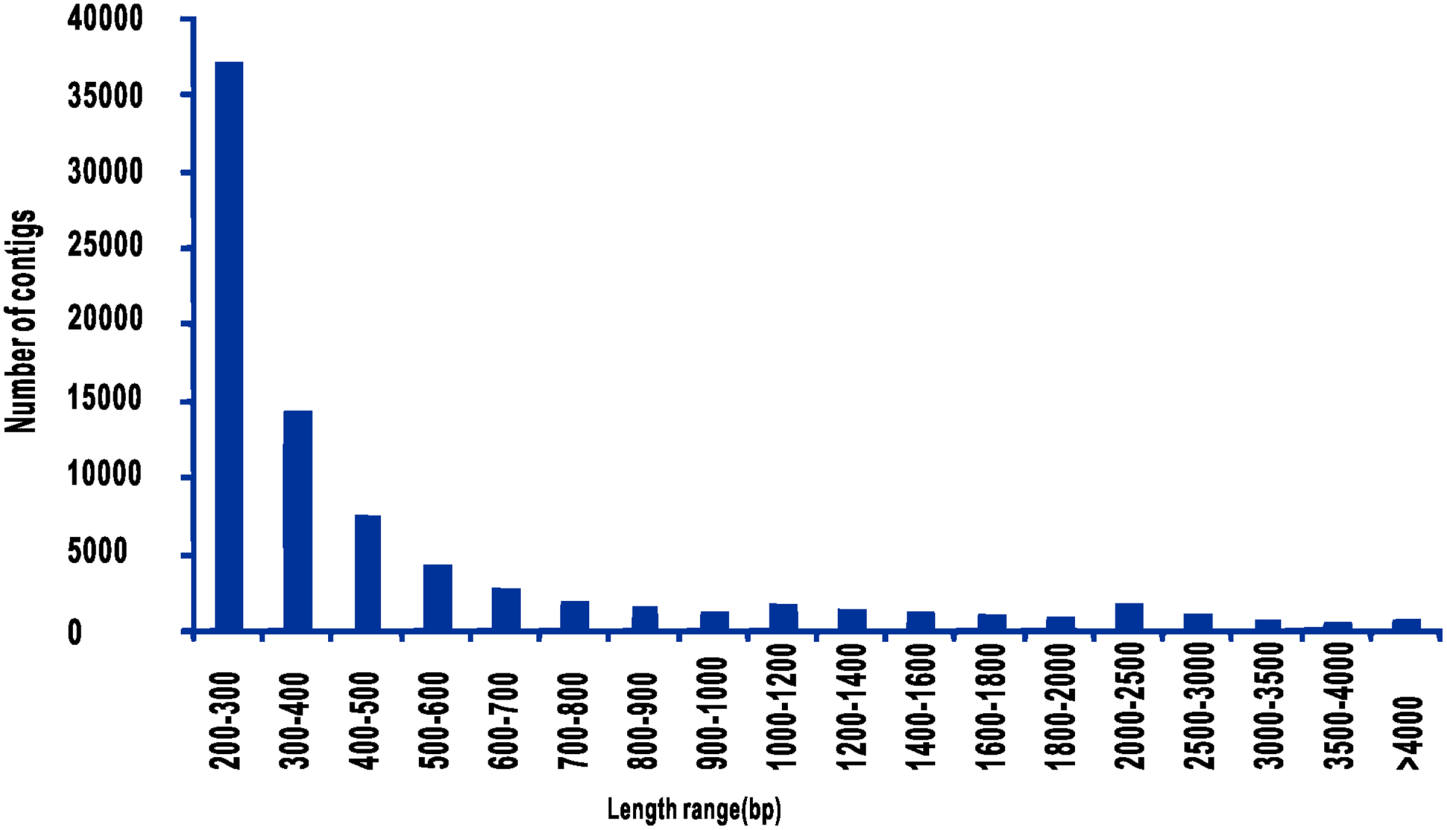
Assembled contig length distribution of the Amur carp transcriptome.

To obtain an overview of the Amur carp gene expression profile, fifteen cDNA samples from different tissues or treatments were prepared and sequenced using an Illumina HiSeq 2000 sequencing instrument. A total of 66.9 Gb of raw data were generated and submitted to NCBI Short Read Archive (SRA) under the accession number of SRP05151. The raw transcriptome data were passed through several quality control filters. After eliminating low-quality bases (quality < 20), sequencing adapters, short reads (< 50 bp) and unpaired reads, a total of 66.3 Gb of high quality reads with an average length of 100 bp were obtained. Clustering and assembly of these reads produced 163,121 contigs with lengths that ranged from 201 bp to 31,440 bp, including 21,516 contigs ≥ 1,000 bp. The mean contig and N50 lengths were 594 bp and 913 bp, respectively. The length distribution of the contigs is shown in Fig 1.

### Annotation and Functional Classification

All assembled contigs were used to predict coding sequences (CDSs) with GETORF from the EMBOSS package [26]. A total of 162,339 CDSs were predicted, and the predicted CDSs were annotated using BlastP with different protein databases including the NCBI non-redundant protein (Nr) database and the UniProt/Swiss-Prot (E-value cut-off of 10^−5^) databases. A total of 32,730 (20.06%) predicted proteins could be annotated with known biological functions, whereas the remaining predicted CDSs were not matched and were annotated as hypothetical proteins (S1 Table).

**Fig 2:**
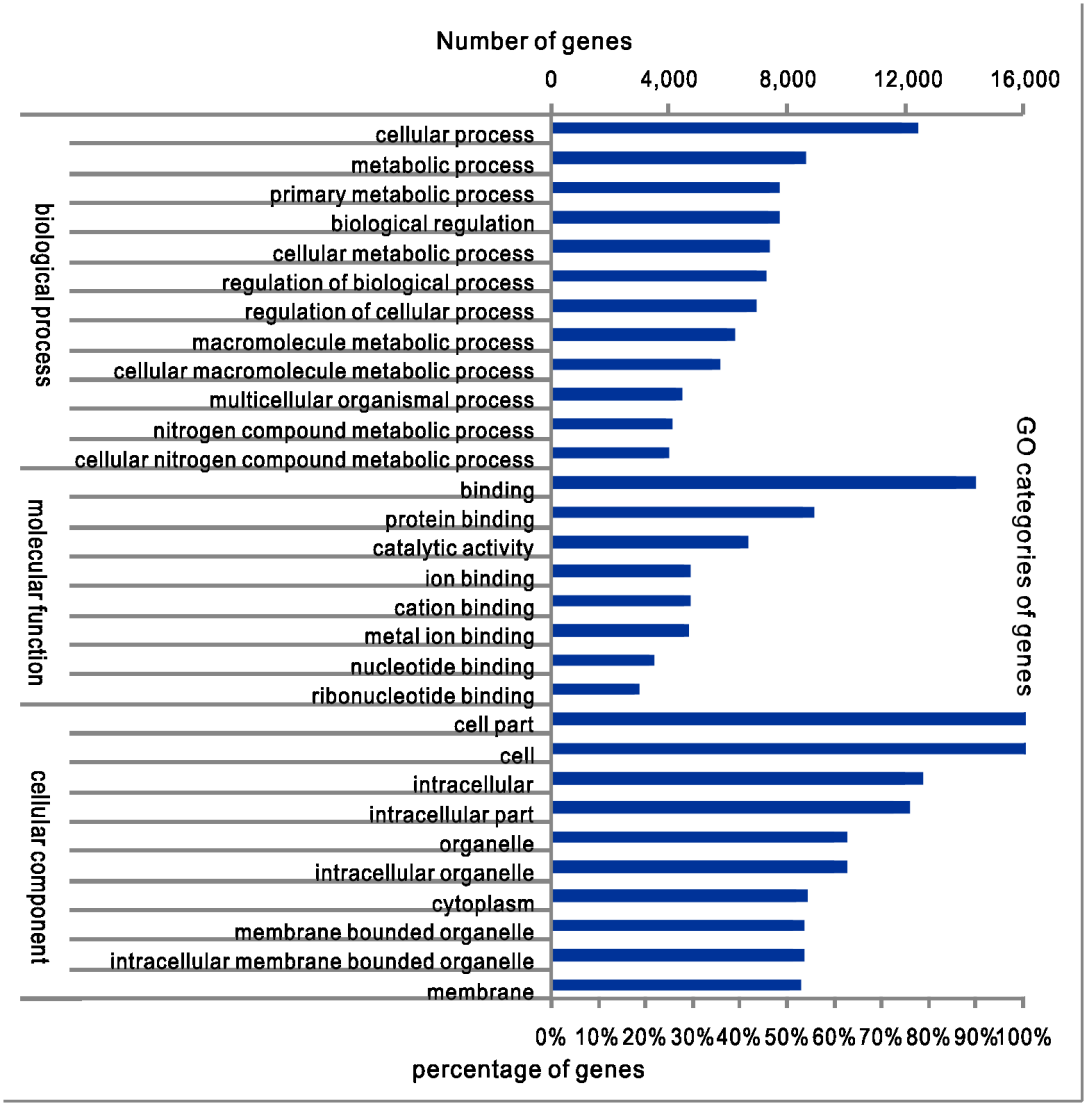
The top 30 most abundant GO sub-categories.

To provide an overview of the different functional classes in the Amur carp transcriptome database, the sequences were also annotated with Gene Ontology (GO) terms. In many cases, one transcript was assigned to multiple terms. Therefore, a total of 19,802 predicted genes were assigned to 132,338 GO terms. There were a total of 7,651 unique GO terms in three categories: biological processes, molecular functions, and cellular components. Of the 19,802 predicted genes, 16,202, 17,388 and 17,830 were assigned to at least one GO term in the biological process, molecular function and cellular component categories, respectively. These sequences were further categorized into 58 primary subcategory functional groups in the three categories (Fig 2).

**Fig 3:**
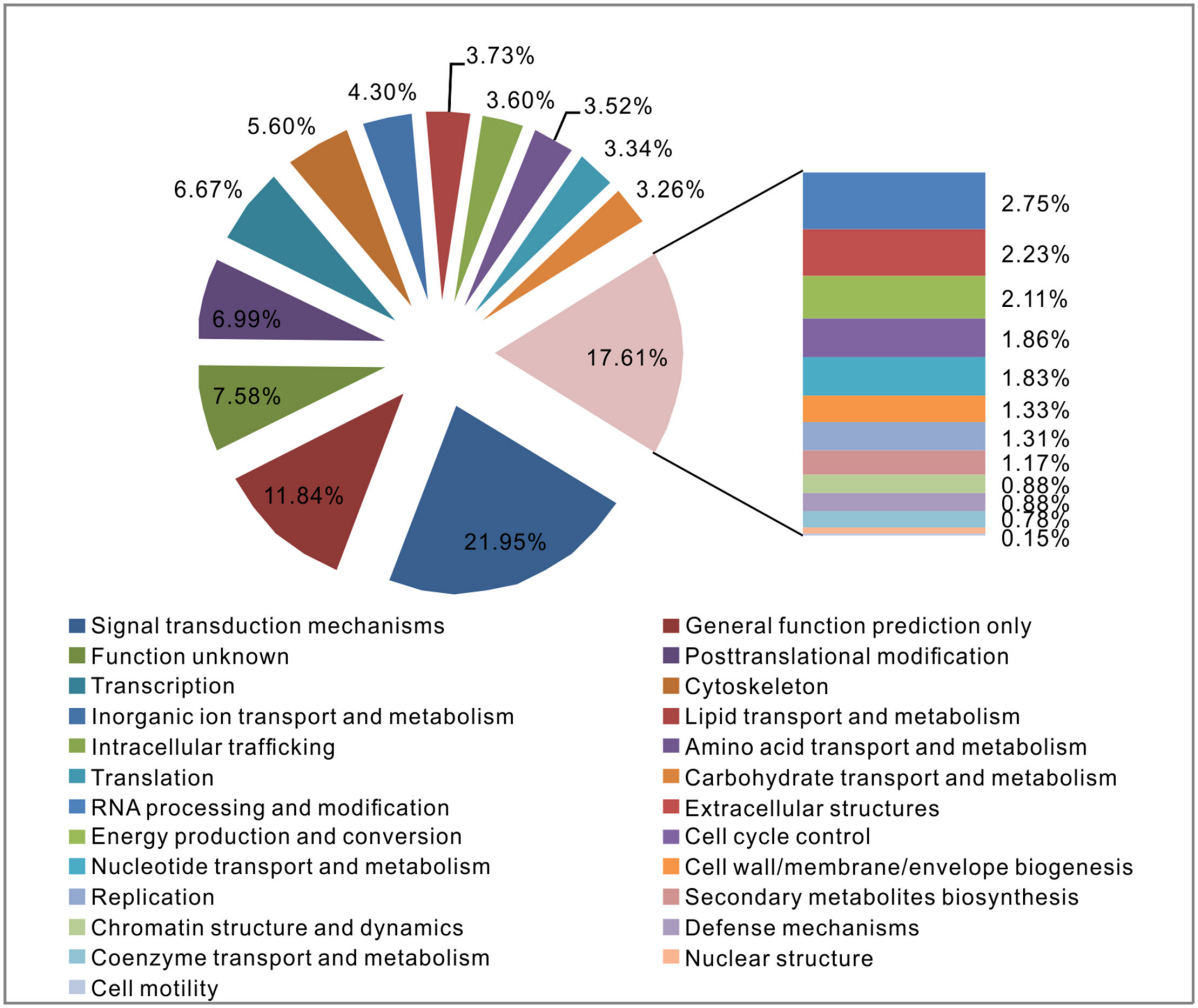
KOG functional classification.

All genes were further annotated and classified based on EuKaryotic Orthologous Groups (KOG) categories. A total of 12,459 genes were annotated and grouped into 25 functional categories (Fig 3). Within these categories, “signal transduction mechanisms” (21.95%) and “general function prediction only” (11.84%) dominated, followed by “post-translational modification” (6.99%) and “transcription” (6.67%). Among the “signal transduction mechanisms,” the most abundant type was the cadherin EGF LAG seven-pass G-type receptor. Additionally, 7.58% of the genes were categorized as “function unknown.”

**Fig 4:**
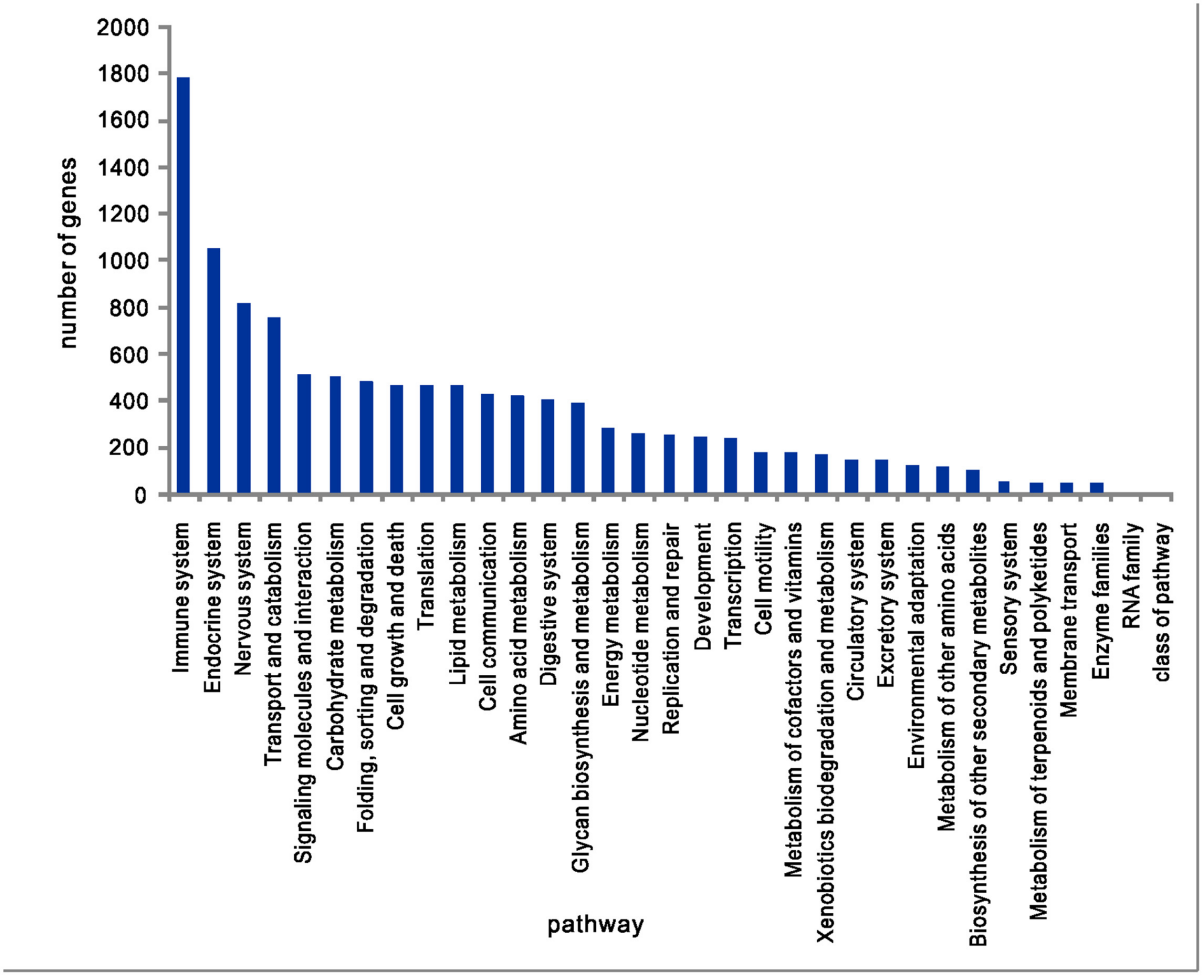
KEGG classification.

KEGG pathway annotations were performed using the KEGG Automatic Annotation Server (KAAS) with the bidirectional best hit information method [27]. The KAAS annotates every submitted sequence with KEGG orthology (KO) identifiers that represent an orthologous group of genes directly linked to an object in the KEGG pathways and the BRITE functional hierarchy [27, 28]. The KEGG pathway analysis revealed diverse pathways with “signal transduction,” “immune system,” “endocrine system,” “nervous system” and “transport and carbohydrate” as the five most highly represented pathways. “PI3K-Akt signaling pathway,” “mitogen-activated protein kinase MAPK signaling pathway,” “neuroactive ligand-receptor interaction,” “endocytosis” and “focal adhesion” were the five most represented subclass pathways (Fig 4).

The signal transduction mechanisms used by fish cells to sense cold signals and trigger intracellular responses remain largely unknown [18]. In this study, some important pathways involved in signal transduction were identified, including the PI3K-Akt signaling, MAPK signaling, calcium signaling, chemokine signaling, and insulin signaling pathways.

### Analysis of Differential Gene Expression and Identification of Cold-Responsive Genes

**Fig 5:**
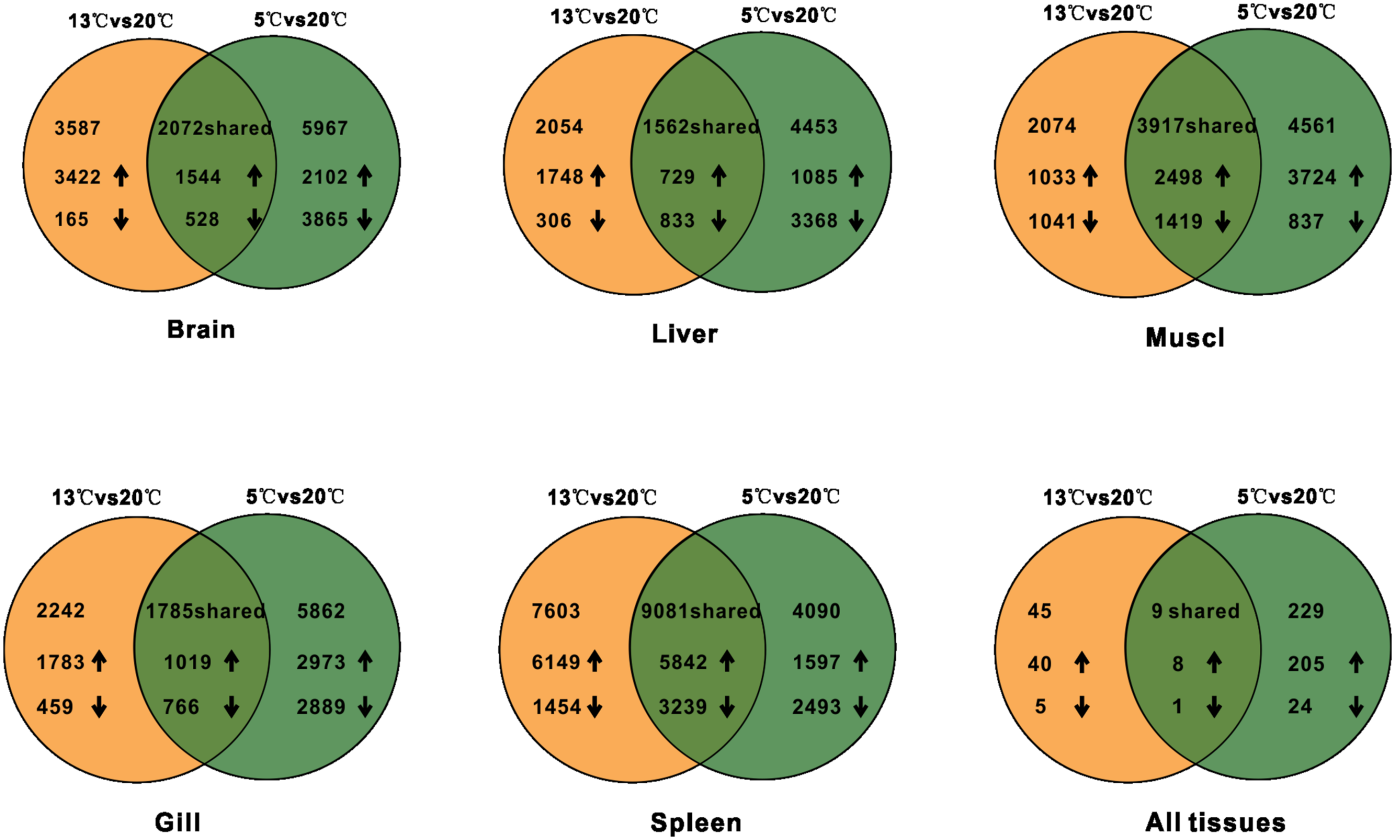
Global gene expression profiles of Amur carp.

Fish adaptation to low temperatures is the result of long-term evolution. Previous studies have shown that common carp can endure a wide range of thermal changes, including daily temperature cycles with ≥ 20°C ranges [7]. To screen for genes associated with cold tolerance, we examined gene expression patterns in five tissues (brain, gill, muscle, liver, and spleen) in Amur carp with long-term acclimation (15 days) to graded cooling (13°C and 5°C); the gene expression profiles in these fish were compared with the expression profiles of fish exposed to the control temperature (20°C) throughout the course of the experiment. The MA-plot-based method with random sampling (MARS) model in DEGseq was used for each tissue [29]. In the cooled fish, the differentially expressed genes that exhibited at least a two-fold change with a false discovery rate (FDR) < 0.001 in at least one tissue were explored. First, we detected changes in gene expression during cold acclimation by comparing fish treated at 13°C and 20°C. After 15 days of the cold challenge at 13°C, a total of 16,684 genes were differentially expressed in the spleen (11,991 upregulated and 4,693 downregulated), followed by 5,991 genes in the muscle (3,531 upregulated and 2,460 downregulated), 5,659 genes in the brain (4,966 upregulated and 693 downregulated), 4,027 genes in the gills (2,802 upregulated and 1,225 downregulated) and 3,616 genes in the liver (2,477 upregulated and 1,139 downregulated). Second, to explore the overall gene expression patterns associated with cold tolerance, we examined the transcript expression in each tissue under severe cold stress (5°C) compared with control conditions (20°C). In these analyses, similar expression profiles were found at 13°C and 20°C, i.e., the spleen contained the highest number of genes with altered expression (7,439 upregulated and 5,732 downregulated), followed by the muscle (6,222 upregulated and 2,256 downregulated), brain (3,646 upregulated and 4,393 downregulated), gills (3,992 upregulated and 3,655 downregulated) and liver (1,814 upregulated and 4,201 downregulated) (Fig 5). Based on the expression profiles of fish at both cold temperatures, we found that genes from the spleen were affected markedly during cold acclimation. The tissue-specific genes that were commonly expressed in both cold-acclimated conditions were used for further analysis.

The differentially expressed genes (DEGs) that were expressed at both 13°C /20°C and 5°C/20°C with fold changes greater than 2 were defined as cold-responsive genes; these genes were detected throughout the course of the experiment. We observed dramatic changes in both the number of DEGs and in the magnitude of gene expression change when comparing of 13°C/20°C and 5°C/20°C. These results are consistent with the life history of Amur carp, which maintains normal physiological and metabolic activities at 13°C and then enters into a dormant state at 5°C with a decrease in the metabolic rate [24]. Additionally, large numbers of genes that were differentially expressed at both 13°C/20°C and 5°C/20°C were detected: 9,081 were identified in the spleen (5,842 upregulated and 3,239 downregulated), followed by 3,917 in the muscle (2,498 upregulated and 1,419 downregulated), 2,072 in the brain (1,544 upregulated and 528 downregulated), 1,785 in the gills (1,019 upregulated and 766 downregulated), and 1,562 in the liver (729 upregulated and 833 downregulated) (Fig 5 and S2 Table). Notably, a large number of immune response-associated genes were highly expressed during long-term acclimation to cold; indeed, decreased immunity is one of the major causes of death of fish at low temperatures [30, 31]. Amur carp survive six months of cold stress, and acquiring strong immunity is presumably an important survival strategy, as indicated by the large number of genes differentially expressed in the spleen during graded cooling in this study.

Although some tissue-specific responses of Amur carp to cold temperatures have been explored, the system-wide integration or whole-body response is poorly understood. To identify the major components of the Amur carp transcriptome data sets that might have a common response to cold, we identified 45 genes that were significantly differentially expressed (40 upregulated and 5 downregulated) in all five tissues between 13°C and 20°C. The number of shared genes increased to 238 (213 upregulated and 25 downregulated) with a decrease in temperature (5°C/20°C), which indicated that most genes associated with cold tolerance in Amur carp are induced at a much lower temperature (5°C) (S3 Table). Fourteen genes that were upregulated at both 13°C and 5°C in different tissues were selected randomly and were confirmed with qPCR (S4 Table and S1 File). At 5°C, which is close to the temperature of the natural habitat of Amur carp during winter (0–4°C), the fish were nearly torpid with a low level of metabolic activity. In a screen for strongly cold-responsive genes, we identified nine differentially expressed genes (eight upregulated and one downregulated) in the five tissues at the two low temperatures (Fig 5) that showed common and/or tissue-wide responses (Table 1); the expression levels of five of these genes were also confirmed with qPCR (S4 Table and S1 File).

**Table 1:** Nine strongly cold-responsive genes found in all five tissues at both 13°C and 5°C.

Seq_name	Annotation	Profile
comp125153_c1_seq1	DnaJ homolog subfamily B member 1	UPa
comp121000_c4_seq1	Protein arginine methyltransferase 1	UP
comp116547_c3_seq1	Hypothetical protein	UP
comp118108_c0_seq1	Inositol-3-phosphate synthase 1-A	UP
comp125362_c7_seq1	LanC-like protein 1	UP
comp126349_c0_seq1	Sialin	UP
comp115203_c5_seq1	Sodium/potassium-transporting ATPase subunit alpha-1	UP
comp77331_c1_seq1	Uncharacterized protein LOC767703 precursor	UP
comp95435_c0_seq1	PREDICTED: otogelin	DOWN

### SSRs Identified in the Amur Carp Transcriptome and in Cold Responsive Genes

Microsatellites, also known as simple sequence repeats (SSRs) or short tandem repeats (STRs), are short repeating segments of 1–6 bp of DNA. Microsatellites are used as molecular markers in genetic studies of linkages in families and in linkage disequilibrium studies of populations. To identify microsatellites in Amur carp, we utilized SciRoKover 3.4 software, which can be used to search for microsatellites throughout the entire genome [32]. A total of 9,427 microsatellite loci with mono-, di-, tri-, tetra-, penta- and hexanucleotide repeats (minimum repeats ≥ 6) were identified in 163,121 contigs. The mean microsatellite frequency was 164.79 SSR markers per Mb. Dinucleotide repeats predominated, with an average ratio of 32.51%, followed by mononucleotide (21.68%) and trinucleotide (20.28%) repeats. Although mono-, di- and trinucleotide repeats comprised the major proportion of SSRs, tetra-, penta- and hexanucleotide repeats, which accounted for 15.30%, 10.23%, and 4.90% of the total SSRs, respectively, were also observed. A/T repeats accounted for approximately 20.34% of the mononucleotide motifs, significantly higher than the C/G repeats (0.69%). Of the dinucleotide motifs, (AC)n repeats were the most common (22.72%), followed by (AG/CT)n (7.48%) and (AT)n (1.37%), whereas perfect and imperfect (GC)n sequence repeats were not found in the Amur carp contigs. (ATC/ATG)n and (AGG)n were the predominant types of trinucleotide repeat, with frequencies of 3.98% and 3.93%, respectively, whereas the other nucleotide repeat motifs had lower frequencies (0.11–3.36%). Of the tetranucleotide SSRs, (AAAT/ATTT)n was the most common (3.64%); the frequencies of the remainder of the nucleotide repeat types were all lower than 3%. There were many types of pentanucleotide and hexanucleotide SSRs, each with low frequencies that ranged from 0.03 to 1.58%. The numbers of mono-, di-, tri-, tetra-, penta-, and hexanucleotide motifs in the different repeat unit classes are listed in Table 2. The average repeat length differed among the various motifs and ranged from 16.7 for mononucleotide motifs to 22.4 for hexanucleotide motifs (Table 2 and S5 Table).

**Table 2:** Characteristics of microsatellites identified in the Amur carp transcriptome.

Motif	Counts	Frequency	Average_Length	Average_Mismatches	Counts/Mbp	GC Content
Mononucleotide	1,948	21.68%	16.7	0.06	34.05	0.04
Dinucleotide	2,922	32.51%	17.78	0.17	51.08	0.48
Trinucleotide	1,823	20.28%	19.05	0.32	31.87	0.44
Tetranucleotide	1,375	15.30%	18.63	0.27	24.04	0.27
Pentanucleotide	919	10.23%	18.14	0.17	16.06	0.3
Hexanucleotide	440	4.90%	22.4	0.26	7.69	0.44
**Frequencies of different motifs**						
AC	2,104	22.72%	17.51	0.14	36.78	0.5
A	1,884	20.34%	16.61	0.06	32.93	0
AG	693	7.48%	18.61	0.26	12.11	0.5
ATC	369	3.98%	19.66	0.37	6.45	0.35
AGG	364	3.93%	19.4	0.38	6.36	0.66
AAAT	337	3.64%	17.81	0.21	5.89	0.01
AGC	311	3.36%	18.63	0.23	5.44	0.66
AAT	237	2.56%	18.91	0.31	4.14	0.01
AAAC	230	2.48%	17.35	0.15	4.02	0.23
AAAG	213	2.30%	18.41	0.22	3.72	0.24
AAG	201	2.17%	18.57	0.3	3.51	0.33
AAC	197	2.13%	18.34	0.24	3.44	0.33
AAAAT	146	1.58%	17.58	0.21	2.55	0
AAAAC	137	1.48%	17.82	0.15	2.39	0.19
ATCC	134	1.45%	20.65	0.54	2.34	0.49
AT	124	1.34%	17.69	0.13	2.17	0
ACAG	108	1.17%	19.16	0.3	1.89	0.49
AAAAG	94	1.02%	18.19	0.17	1.64	0.19

As mentioned above, numerous SSRs of various types were found at high frequencies in the Amur carp transcriptome. These SSRs provide abundant candidate molecular markers for genetic diversity analysis, genetic mapping and molecular breeding applications in this species. To identify SSRs probably having important regular roles in cold-responsive genes, a large number of SSRs in tissue-specific cold-responsive genes were detected. There were 1028 SSRs in spleen, 450 in muscle, 204 in brain, 144 in gill and 126 in liver tissues (S6 Table). These SSR markers will be useful for identifying cold tolerance candidate genes using population genetic methods, and they will play important roles as molecular markers in the breeding of common carp with cold tolerance in the future.

### GO Enrichment Analysis of Cold-Responsive Genes

**Fig 6:**
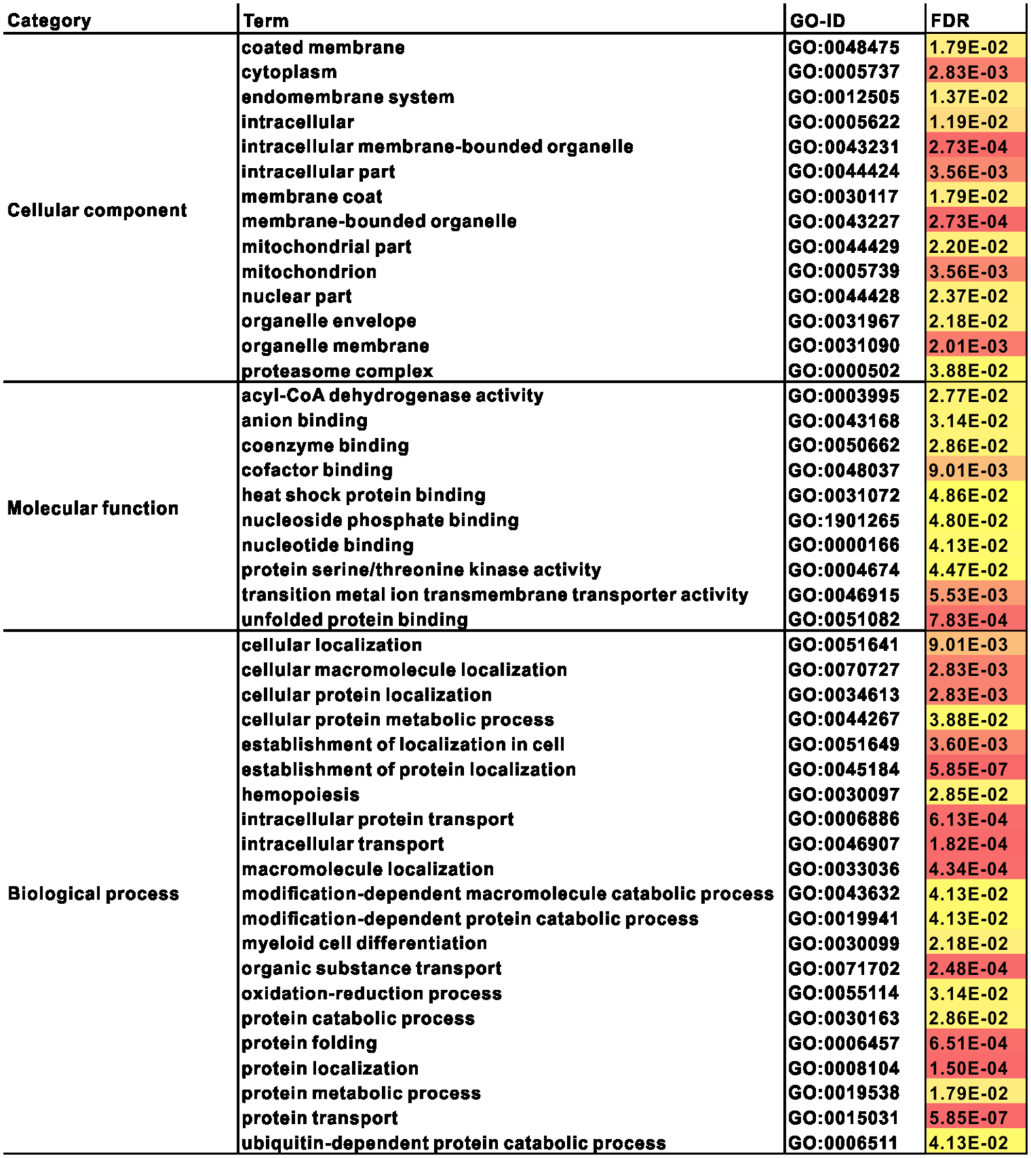
Gene ontology (GO) enrichment analysis of cold-responsive genes.

In this study, we focused on cold-responsive genes that were differentially expressed in at least one tissue. GO term enrichment of differentially expressed genes from each tissue dataset was performed using Fisher’s exact test (p-value < 0.001) through Blast2Go. A list of the significantly enriched GO terms of the DEGs in at least one tissue during a stepped cooling regime is shown in S7 Table. Fourteen enriched subcellular structures (p < 0.001) were identified in the cellular compartment analysis, including the cytoplasm, intracellular membrane-bounded organelles, membrane-bounded organelles, mitochondria, organelle membranes, and intracellular and coated membranes. The GO molecular function indicated that ten categories had significantly higher enrichment (p < 0.001) compared with the total set of differentially expressed transcripts, and the categories “unfolded protein binding,” “transition metal ion transmembrane transporter activity,” and “cofactor binding” were ranked highest, suggesting prominent biological functions. The GO biological process detected 21 categories and these biological pathways included not only protein-related processes (protein transport, protein folding, protein metabolic processes, protein catabolic processes, and establishment of protein localization) but also cellular and extracellular structure-related processes (cellular localization, cellular macromolecule localization, cellular protein localization, cellular protein metabolic processes, intracellular transport, intracellular protein transport, macromolecule localization and organic substance transport) (Fig 6).

In this study, “protein localization” and “protein transport” showed the highest enrichment. GO terms that represented the differentially expressed genes involved in mitochondrial and membrane metabolism were also enriched. Several lines of evidence indicated that both mitochondrial transcripts [33] and the number of mitochondria were increased in the skeletal muscle of some cooled fish. Moreover, an increase in the number of mitochondria in cells is typically associated with an increase in cytochrome oxidase activity; indeed, cytochrome oxidase activity in fish tissues increases to compensate for low temperatures [33, 34]. Our findings are consistent with the above observations. Two GO terms related to mitochondria and a large number of genes related to cytochrome, which was markedly upregulated during temperature changes, were enriched in Amur carp. These results are similar to those obtained by Gracey et al (2004). Moreover, many of the highly overrepresented functional GO categories of cold-induced genes from this study were also found in Gracey et al.’s study of carp exposed to cold stress [13]. Although, we assessed system-wide integration and whole-body responses, we did not identify any genes that were enriched in all the tissues.

### Pathway Enrichment Analysis of Cold-Responsive Genes

**Fig 7:**
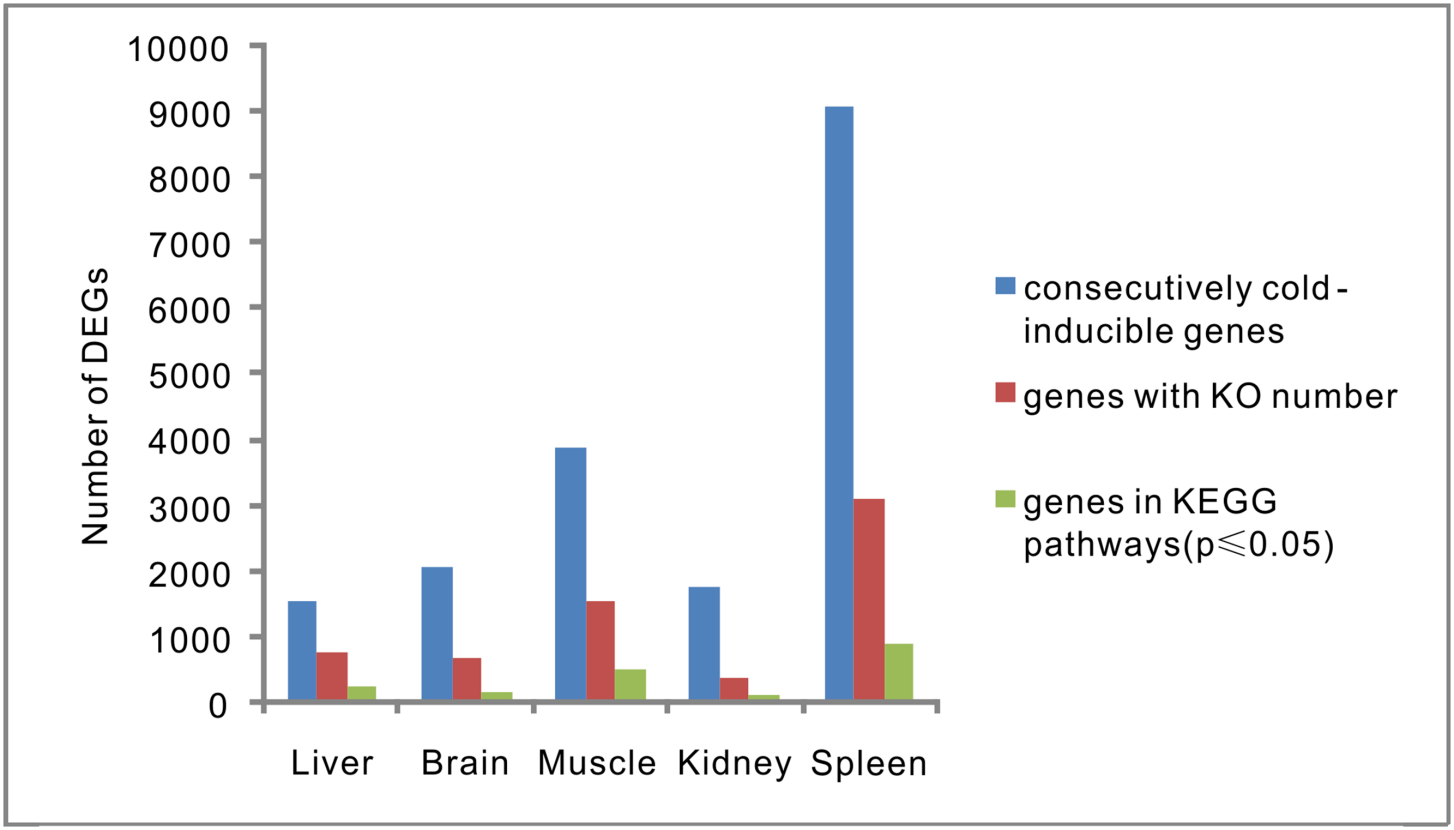
Distribution of cold-responsive genes in each tissue.

**Fig 8:**
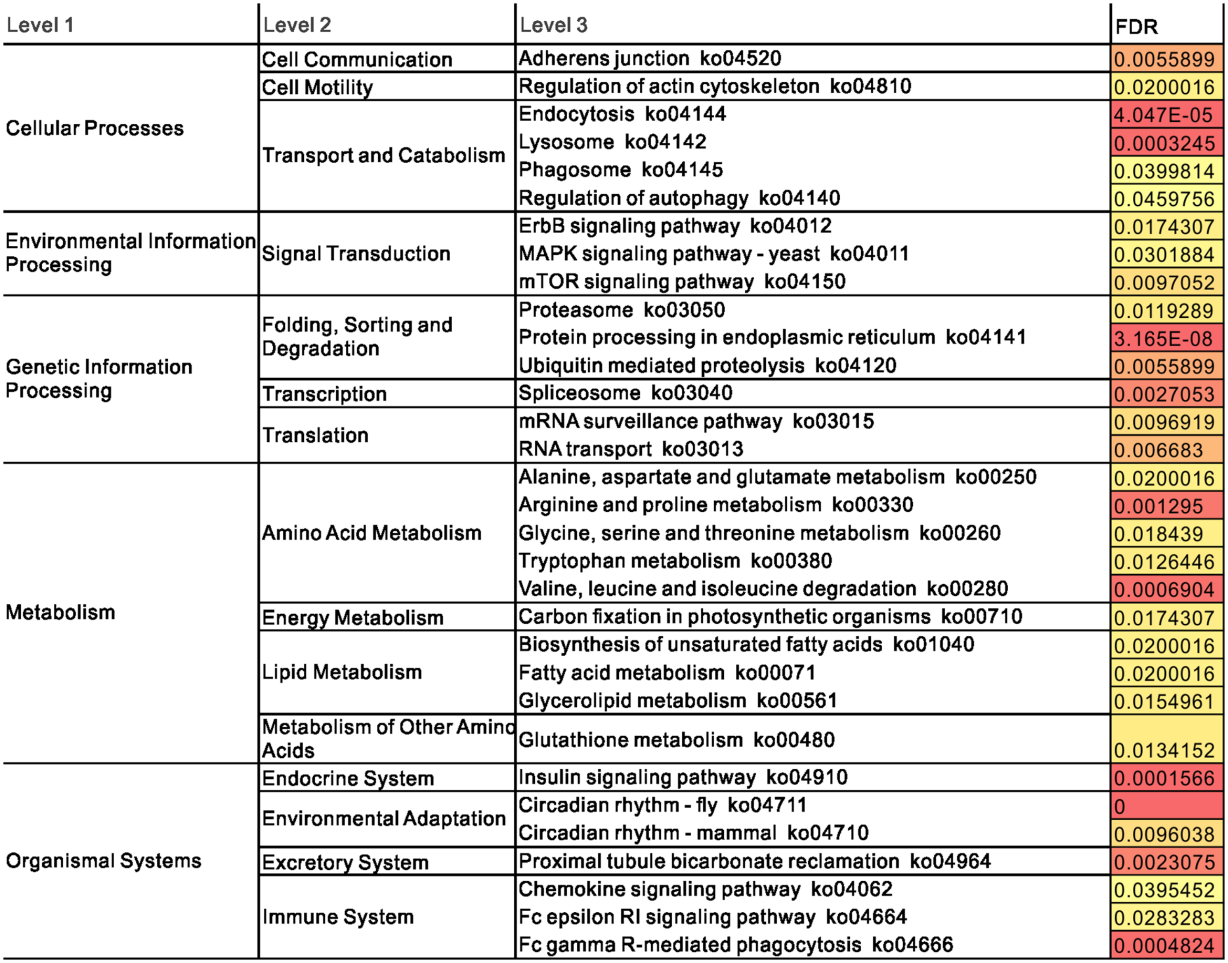
Pathway enrichment analysis of cold-responsive genes.

To identify the primary biochemical pathways involved in the cold response, we performed metabolic pathway enrichment analysis by comparing the cold-responsive genes with the total set of differentially expressed transcripts. A total of 1,953 transcripts in the KEGG pathway were found to be involved in adaptation to cold. The following analyses and discussions of the metabolic pathways are based on these transcripts, and detailed information about the number of transcripts in each tissue is provided in Fig 7. The identified genes were related to 32 metabolic pathways that changed significantly under cold stress (p ≤ 0.05), including genes involved in “cellular processes (cell communication, cell motility, and transport and catabolism),” “environmental information processing (signal transduction),” “genetic information processing (folding, sorting and degradation, transcription, and translation),” “metabolism (amino acid metabolism, energy metabolism, lipid metabolism, and metabolism of other amino acids),” and “organismal systems (endocrine system, environmental adaptation, excretory system, and immune system)” (Fig 8). Many of the overrepresented functional pathways of cold-responsive genes from this study overlapped with those found in all analyzed tissues of carp exposed to cold stress [18, 19]. Pathways such as “circadian rhythm,” “spliceosome,” “proteasome,” “MAPK signaling pathway,” “mTOR signaling pathway,” and “biosynthesis of unsaturated fatty acids” were enriched among the cold-responsive genes in previous studies and in our current study.

Based on the normalized read counts of each mRNA in the control and cold libraries, we deduced the gene expression changes in response to cold acclimation in at least in one tissue. To obtain a clear understanding of the system-wide integration of responses, all differentially expressed genes in all five tissues were combined. From this dataset, we obtained 40 common upregulated genes in mild cold (13°C/20°C) and 213 common upregulated genes in severe cold (5°C/20°C) (S3 Table). To discover enriched functionally related gene groups and cluster redundant annotation terms, all differentially expressed genes were mapped to terms in the KEGG database and were then compared with the entire transcriptome background. None of the genes in any of the pathways were enriched under mild cold conditions (13°C), whereas under severe cold stress (5°C), we found overrepresentation of two KEGG pathways in the genes upregulated by cold stress, “spliceosome” (KO03040) and “protein processing in endoplasmic reticulum” (KO04141). In the study of Yong Long et al., the “spliceosome” was the most significantly enriched pathway in zebrafish under cold acclimation [18, 19]. We also found that “spliceosome” was the most significantly enriched pathway, with 15 genes upregulated. There were 12 upregulated genes enriched in “protein processing in endoplasmic reticulum”. Common carp and zebrafish are in the same cyprinid family of fish; thus, the highly overrepresented pathways of cold-induced genes from carp in this study overlapped with those found in all the analyzed tissues of zebrafish exposed to cold stress [13].

## Conclusions

In this study, the Amur carp transcriptome was sequenced using the HiSeq2000 platform and subsequently assembled. Unique CDSs were identified and functionally annotated via comparisons with different protein databases. A large number of cold-responsive genes were detected in different tissues at different temperatures. Additionally, cDNA SSR loci associated with cold tolerance were identified for future marker development and genetic analyses. GO enrichment and pathway enrichment analyses of cold-responsive genes were conducted, revealing that Amur carp has evolved special strategies to survive low temperatures.

## Materials and Methods

### Ethics Statement

The Animal Care and Use Committee of the Heilongjiang River Fisheries Research Institute, Chinese Academy of Fishery Sciences, approved this study.

### Experimental Fish and the Cold Challenge Experiment

With the approval of the Fisheries Bureau of Heilongjiang Province, wild Amur carp were caught from the Amur River in China and were reared at the Hulan Experimental Station of the Heilongjiang River Fisheries Research Institute, Chinese Academy of Fishery Sciences. Three-month-old fingerlings produced by a single mating pair of wild Amur carp were selected for this experiment. The fingerlings (mean weight of 150 g) were maintained in 200-L tanks with circulating aerated water at 20°C for one month. For cooling, all fish were subjected to a stepped cooling regime of 1°C/h for a maximum of 7°C/day. The fish were cooled from 20°C to 13°C and then acclimated at 13°C for 15 days before sampling. The remaining fish were cooled continuously from 13°C to 5°C with the same rate of cooling and were acclimated at 5°C for an additional 15 days before sampling. The 20°C-acclimated (control) fish were sampled at the end of the cooling experiment. After exposure to temperature stress, the fish were anesthetized with 0.016% tricaine. Samples were collected from five tissues, the brain, liver, spleen, gills, and muscle of three individuals from the same temperature group and were stored in RNAlater stabilization reagent (Ambion, Austen, TX, USA). For Illumina sequencing, the same tissues from three individuals from the same temperature group were mixed equally. For the qPCR, three tissues (liver, gill and spleen) were analyzed from the same individuals used for sequencing in each group.

### RNA Extraction and mRNA Purification

Each frozen sample was ground in a mortar with liquid nitrogen. Total RNA was isolated using TRIzol reagent (Invitrogen, Carlsbad, CA, USA) following the standard protocol. The obtained total RNA was dissolved in 200μL of RNase-free water. The concentration of total RNA was determined with a NanoDrop spectrophotometer (Thermo Scientific, South Logan, UT, USA), and the RNA integrity value (RIN) was determined with an RNA 6000 Pico LabChip kit and an Agilent 2100 Bioanalyzer (Agilent Technologies, Santa Clara, CA, USA).

Total RNA was incubated with 10 U of DNase I at 37°C for 1 h; then, nuclease-free water was added to bring the sample volume to 250 μL. The mRNA was further purified with the MicroPoly (A) Purist Kit (Ambion) following the manufacturer’s protocol. The mRNA was dissolved in 100 μL of RNA Storage Solution. The final concentration was determined with a NanoDrop spectrophotometer.

### cDNA Preparation and Sequencing

The mRNA was fragmented and converted into an RNA-seq library using the mRNAseq library construction kit (Illumina, San Diego, CA, USA) according to the manufacturer’s instructions. We performed 2x100 bp paired-end sequencing with the Illumina HiSeq2000 (Illumina, San Diego, CA, USA). All sequenced reads have been uploaded to the NCBI SRA database under the accession number of SRP05151.

### Sequence Assembly

The sequence reads from all samples were cleaned using the FASTX toolkit (http://hannonlab.cshl.edu/fastx_toolkit/). First, all reads containing 'N' were discarded using a Perl script; subsequently, adapter sequences were removed using the fastx_clipper program, which was followed by removal of low quality (Q < 5) bases from the 3’ end with fastq_quality_trimmer, which required a minimum sequence length of 50 bp. Finally, the reads in which at least 90% of the bases had a base quality > 20 were filtered using fastq_quality_filter for further assembly. *De novo* assembly of the transcriptome was performed using the Trinity RNA-Seq assembly v2013-02-25 with default parameters [PMID: 21572440].

### Bioinformatics Analysis

The open reading frames were identified using an in-house program developed based on “GetORF” from EMBOSS [26]. Gene annotation was performed via a BlastP search against the Swiss-Prot and GenBank databases with an E-value of 1e^-5^. Next, we chose the best single result for the gene annotation. Gene ontology analysis was performed using GoPipe [35] through BlastP against the Swiss-Prot and TrEMBL databases with an E-values of 1e^-5^. The metabolic pathways were constructed based on the KEGG database using the bidirectional best hit (BBH) method [36]. The analysis first provided the KO number for each protein; then, the metabolic pathways were constructed based on the KO number.

The read number of each contig was first transformed into reads per kilobase per million reads (RPKM) [37] before the identification of differentially expressed contigs with the DEGseq package using the MARS [30]. We used the “FDR ≤ 0.001 and the absolute value of log2Ratio ≥ 1” as the thresholds to determine significant differences in contig expression.

### Real-Time qPCR Analysis

cDNA from each sample was synthesized using the PrimeScript RT Reagent Kit with gDNA Eraser (TaKaRa, Dalian, Liaoning, China), according to the manufacturer’s protocol. The gene-specific primers are listed in S4 Table. SYBR Premix Ex Taq II (TaKaRa) was used to quantify the expression of each target gene using an ABI 7500 quantitative PCR machine (Applied Biosystems, Foster City, CA, USA). A two-step RT-PCR program was performed as follows: an enzyme activation step at 95°C for 10 min followed by 40 cycles of 95°C for 15 s and 60°C for 60 s; 18S ribosomal RNA served as an internal control [38]. The data were collected and analyzed with the 2^(-ΔΔCt)^ method [39].

## Supporting Information

S1 File Expression levels of fourteen selected genes in three tissues as determined by qPCR. (PDF)

S1 Table Amur carp gene annotations. (XLSX)

S2 Table Cold-responsive genes in each tissue under two temperature conditions (13°C and 5°C). (XLS)

S3 Table Genes exhibiting a common response in all tissues under two temperature conditions (13°C and 5°C). (XLS)

S4 Table Sequences of primes used for qPCR amplification of fourteen genes. (XLS)

S5 Table SSRs identified in the Amur carp transcriptome. (XLS)

S6 Table SSRs identified in cold-responsive genes. (XLS)

S7 Table Gene ontology (GO) enrichment analysis of cold-responsive genes. (XLS)
