# Supplementary figures and images for: Transcriptome Analysis to Identify Cold-Responsive Genes in Amur Carp (Cyprinus carpio haematopterus)
Source: PLoS One. 2015 Jun 22;10(6):e0130526. doi: 10.1371/journal.pone.0130526 (PMC4476670; doi:10.1371/journal.pone.0130526)

S1 File. Expression levels of fourteen selected genes in three tissues as determined by qPCR

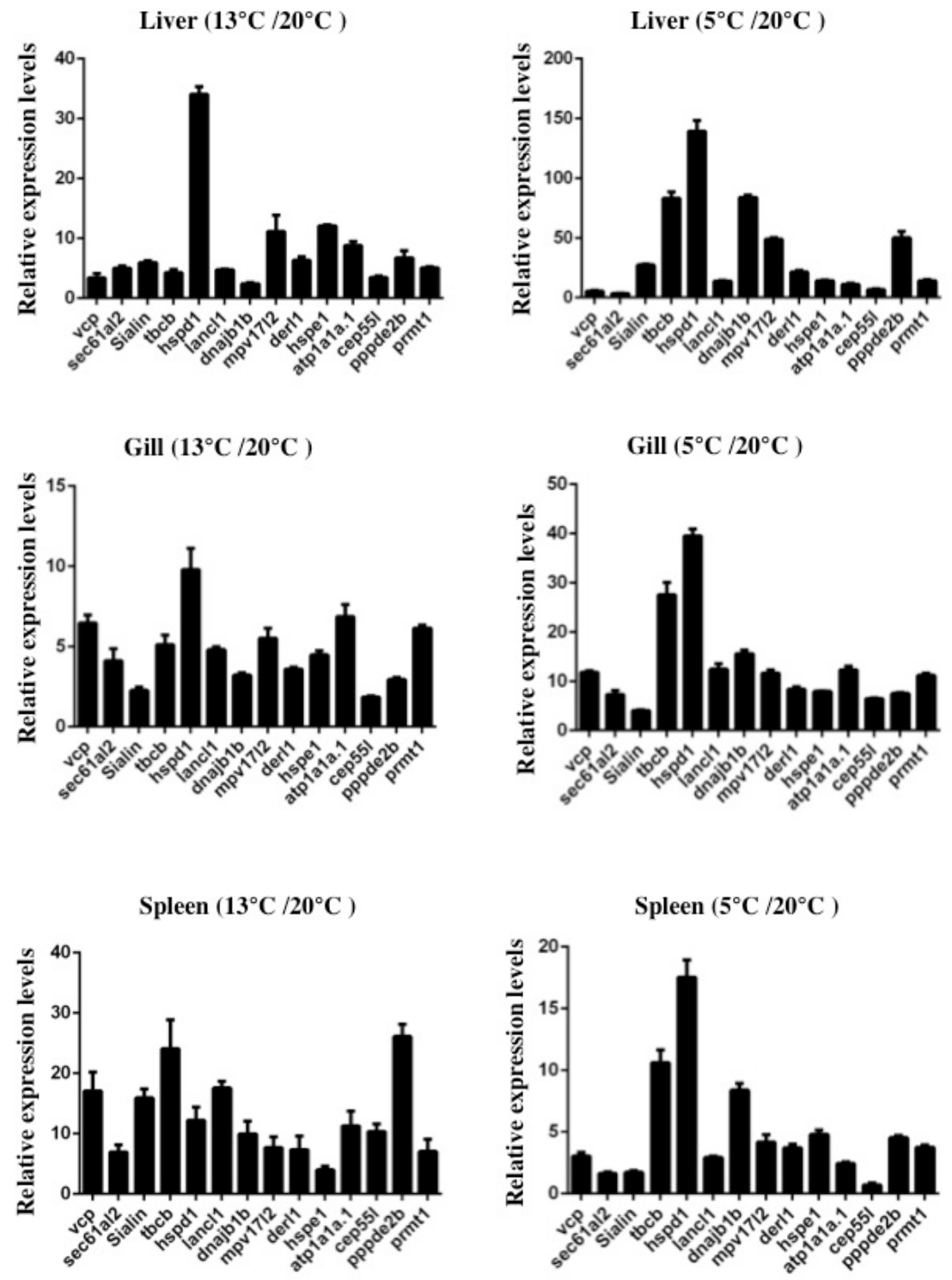

Supplement: S1 File — (PDF) [file pone.0130526.s001.pdf]
